# Supplementary figures and images for: Decreasing death rates and causes of death in Icelandic children—A longitudinal analysis
Source: PLoS One. 2021 Sep 30;16(9):e0257536. doi: 10.1371/journal.pone.0257536 (PMC8483359; doi:10.1371/journal.pone.0257536)

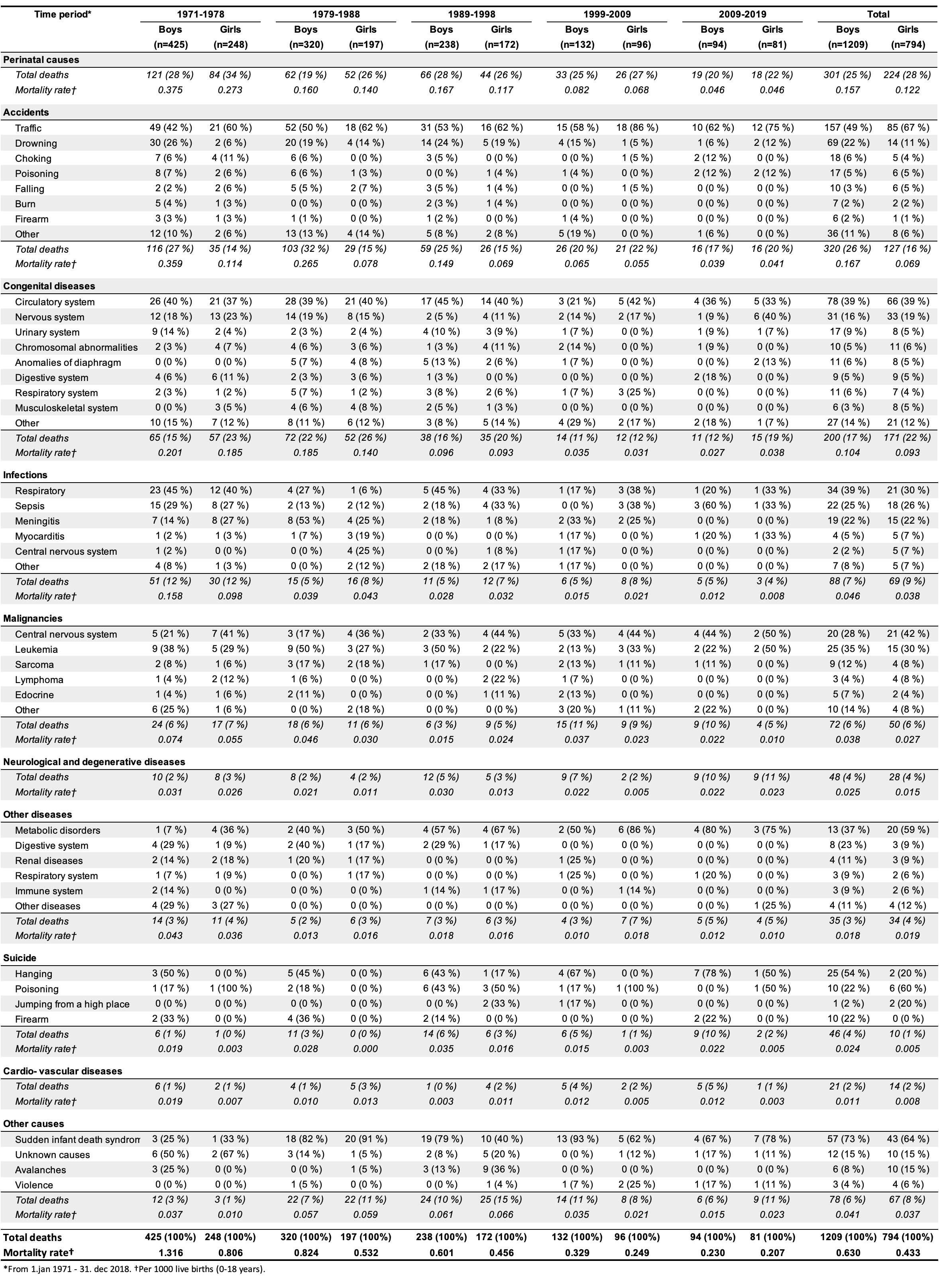

Supplement: S1 Table — (DOCX) [file pone.0257536.s001.docx]
