# Supplementary material for: Decreasing death rates and causes of death in Icelandic children—A longitudinal analysis
Source: PLoS One. 2021 Sep 30;16(9):e0257536. doi: 10.1371/journal.pone.0257536 (PMC8483359; doi:10.1371/journal.pone.0257536)
Supplement: Attachment — Submitted filename: PLOS-response to revieweres.docx [file pone.0257536.s002.docx]

**Response to the reviewers**

Let us start by thanking the revivers for their reviewing of the manuscript and several interesting comments and suggestions. We have complied to the best of our capability with their suggestions and find the manuscript improved.

We do hope that our responses provide sufficient answers and adaptations to the manuscript.

5. Review Comments to the Author

Please use the space provided to explain your answers to the questions above. You may also include additional comments for the author, including concerns about dual publication, research ethics, or publication ethics. (Please upload your review as an attachment if it exceeds 20,000 characters)

Reviewer #1: In the manuscript “Decreasing death rates and causes of death in Icelandic children—A longitudinal analysis”, the authors analyze a near 50 year dataset of mortality data to ascertain how death rates have changed over the study period. More specifically, the authors subset the data into sex and age groups, calculate mortality rates for various causes of death, and compare how and/or model how how rates have changed over the period. This is an interesting topic and use of public health data, as it has the potential to evaluate the affect of past public health policies. However, several major aspects of the current paper prevents me from recommending it for publication in this journal.

First, the trove of data the authors use is certainly worth analysis, and Figures 1 and 2 are interesting in and of themselves. However, the authors do not establish any sort of questions or hypotheses that they wish to answer or evaluate by doing the analysis. Thus, it feels more like a preliminary data exploration exercise than a mature, hypothesis driven analysis. There is an implicit hypothesis, captured in one of the statistical methods used (Chi-square), that the mortality rates at the end of the study period differ from those at the beginning. However, the lack of explicit questions or hypotheses results in a disappointingly short exploration of some of the more surprising results, such as the marginal increase in death rates due to suicide in recent decades. I would recommend re-examining the whole of the data, and more thoroughly considering which patterns are expected versus ones that are not, and thus suggest the need for re-evaluation of public health policy.

We thank the reviewer for these general and important comments and for the thorough reviewing of the manuscript. We have adapted the manuscript accordingly. The following has been added to the introduction section:

In the current study we investigated the main hypothesis that an important decrease in the childhood death rates in Iceland during almost fifty years was primarily driven by fewer fatal infections in young children and childhood accidents and lower neonatal mortality.

The final paragraph of the introduction has been adapted to the reviewer’s comment (see also minor comment/Introduction and our reply):

The aim of the current study was to describe changes in childhood death rates in Iceland and evaluate causes of death over almost five decades. The underlying hypothesis was that death rates are declining, especially the under-five mortality rate. From the results we attempt to determine specific risk factors and identify potential interventions to further prevent childhood deaths.

We do share the reviewer’s thoughts on “… the marginal increase in death rates due to suicide in recent decades.” This is truly of grave concern and although the increase is not significant, all other causes show decreasing death rates. We have added to the paragraph on suicidal death in the discussion, the whole paragraph currently reads as follows:

The rate of children dying due to suicide did not change significantly during the study period. Similar findings have been reported by others^29,30^ although other Nordic countries have reported a decreasing incidence.^31^ As in other studies, boys are at much higher risk than girls.^29–31^ This finding is of a grave concern. We recognise that determining whether a cause of death should be categorised as suicide is often difficult. The available data may not always be sufficient to distinguish between accidents e.g. by drug overdose and suicides. Suicide may therefore possibly be underestimated, especially in teenagers. However, heightened awareness of suicidal risk in teenagers is important for implementing preventive measures. This finding of no or slim reductions in death rates due to suicide in our study must be a strong encouragement to increase our awareness of the importance of mental health and psychological disorders in children in order to prevent these tragic events. A working group on suicide preventions issued by the Icelandic government released a program of action in 2018 with one of the goals being to ensure regular and evidence-based education on mental health in day-care centres, as well as primary and secondary schools. This, among other measures, will hopefully lead to better awareness and bring us one step closer to intervene and provide help to those who need it before it is too late.

Second, the statistical methods chosen seem reasonable given the data, but how the data were processed, how rates were calculated, and how the statistical methods were applied to the data are very much unclear. I was also frequently uncertain whether the presented results pertained to the Chi-square or the Poisson regression analyses. Further, some of the results discussed in the text did not seem to align with the data in the tables. For example, in paragraph 1 of the results, it says “The death rate decreased significantly over the study period, for boys the ratio decreased from 1·5:1000 boys to 0·18 and for girls from 0·9 to 0·17:1000 girls Table I)”. However, the table shows different values for boys and girls in the first and last decade examined. I suppose this sentence was referring to the first and last years, but this isn't clear. And, the figure does not differentiate between girls and boys, so that doesn't help clarify things. To evaluate the rigor of a study, it is imperative that statistical methodologies are clearly explained, and that there is an unambiguous connection between analyses conducted and results presented.

For better clarification of the statistical methods, changes have been made to the methods section (see also later).

For the sentence in question the following adaptations have been made:
“The death rate decreased significantly over the study period. For boys the ratio decreased from 1·5:1000 in 1971 to 0·18 in 2018 and for girls from 0·9 to 0·17:1000 girls in the same years (Table I)”.

Next, the results are presented in a “laundry-list” sort of manner that may be more appropriate for a journal of public health statistics. In addition, organization of the results section into subsections would significantly improve the readability of that section.

We do not quite understand this comment. The results section is short, the tables in sections and the figures descriptive. There are only five paragraphs in this section, and we are not convinced that braking this up into sub-sections would be helpful. Nevertheless, we made some rearrangements of the text for clarification and did split the 5^th^ paragraph into several paragraphs. We do agree that this improves the section and clarifies some issues.

Lastly, the discussion section does discuss results in the context of other HIC, and some reasonable interpretations for how past interventions could have resulted in the patterns shown are made. However, it does not “determine specific risk factors and analyse possible interventions” as suggested in the introduction. The determination of risk factors and determining important contributors to mortality rates could be done via appropriate multivariate modeling. This is not done here, as I far as I can tell. In addition, the discussion of interventions for some causes of mortality, such as from accidents, are discussed only vaguely and lack citations.

We thank the reviewer for pointing out that more discussion is justified. We have added some more comments and discussion to this section and added one reference.

The reviewer comments that the discussion “does not determine specific risk factors and analyse possible interventions” as suggested in the introduction.” We have indeed in this section addressed factors such as new antibiotics and improved antibiotic usage, high vaccine coverage, improved perinatal health care, improved prenatal diagnostics, better treatment for malignancies, accident prevention and heightened awareness for accidents. Nevertheless, we agree that these factors may be highlighted even more clearly and have made adjustments to the Discussion section.

The suggestion on “appropriate multivariate modelling” was indeed discussed in our group. However, in this study of death rates over a period of fifty years this is complicated and may even be misleading. During this period, a vast number of interventions, new treatment options, laws and regulations for traffic safety, improved diagnostics etc have been implemented. In addition, these factors weigh differently on the various causes of death and changes over time. It was therefore decided against using multivariate analysis. Nevertheless, the text in the discussion has been adapted for further clarification as pointed out by the reviewer.

Several adaptations have been made to the discussion. The second paragraph of the discussion now reads as follows:

The gender ratio clearly revealed higher risk of death for boys than girls, most obvious in the adolescent age group and more pronounced in the first decades of the study period. These ratios have been described by others.^9,10^ This gender difference is less clear in under five mortality rates as has also been described by UNICEF in HIC.^1^ In our study, a decline in male/female ratio was seen from 1·6 to 1·2 and deserves special attention. The reason may be due to behavioural differences as well as physiological, social and environmental factors. This is reflected in the higher death rate for boys due to accidents. The decreasing gender ratio was in fact primarily driven by fewer fatal accidents among boys. These causes have been identified in several countries^8^. During the study period, several safety measures for accident prevention were implemented, especially regarding traffic safety. These safety measures as well as increased awareness are probably the main factors leading to the decreased childhood mortality due to accidents.

One reference has been added:

Peden M, Oyegbite K, Ozanne-Smith J, et al. World report on child injury prevention. Geneva: World Health Organization; 2008.

In the third paragraph perinatal health care is discussed. To this paragraph we have added the following:

Perinatal and neonatal deaths in Iceland have been substantially decreasing over the period and is currently among the lowest in Europe^12^. This obviously contributes to the improved survival in this age group.

The chapter on suicide has already been mentioned and has been adapted to the reviewer’s suggestions.

Minor comments: there are no line numbers on the PDF of the submission. This makes it difficult to refer to specific sections. Therefore, I will refer to paragraphs of sections.

Abstract: The word “impressive” in inappropriate here, as it has an emotional connotation.

The word “impressive” has been replaced with “distinct”.

Findings section: what does “a less impressive reductions was seen in other causes” mean?

We changed the two sentences according to this comment. It now reads:

This reduction was primarily attributed to a decrease in fatal accidents and fewer deaths due to infections, perinatal or congenital disease as well as malignancies, the reduction in death rates from other causes was less distinct. Childhood suicide rates remained constant.

Introduction: the last paragraph is a single sentence. It should be split into several sentences for clarity.

The paragraph now reads as follows (see also our reply to an earlier comment):
The aim of the current study was to describe changes in childhood death rates in Iceland and evaluate causes of death over almost five decades. The underlying hypothesis was that death rates are declining, especially the under-five mortality rate. From the results we attempt to determine specific risk factors and identify potential interventions to further prevent childhood deaths.

Methods:
1st paragraph: causes of death weren't described in this study, they were compiled from records.

This currently reads:

In the current study, causes of death in children (from birth until their 18th birthday) were collected from The National Causes of Death register in Iceland.

2nd paragraph: What is the ICD-8 through 10 system? Is it a relevant detail here?
We find this relevant for clarification of the methods and diagnosis and for comparison with other studies.

1. In the case of “ill-defined and unspecified causes of mortality”, how were hospital records used to assign cause of death? Was this done in a consistent way by a qualified person? Is there uncertainty in this process, and what proportion of the data is made up of this classification

The National Causes of Death register in Iceland is governed by the Directorate of Health in Iceland and coded according to international standards by trained persons. This gave us the cause of death in all but 57 of the 2003 cases identified. When cause of death was coded as “ill-defined and unspecified causes of mortality” (ICD-10: R99), two of the authors (MRL and ÁH) looked up hospital records to find if these data revealed the actual cause of death. If the cause was still unclear, the corresponding author (ÁH) consulted the head of Pathology at the University Hospital to find out if autopsy reports revealed the cause of death. If the cause of death was still unclear the child was put into the category of “Other causes: Unknown causes” (n=22).

It is unclear to us how much should be added to the text to clarify this. We find the question if this was done by a “qualified person” unusual. Nevertheless, we have made the following adaptations:

Information was gathered from The National Causes of Death register in Iceland, kept at the Directorate of Health, Iceland and maintained by qualified personnel.

If cause of death was classified as “other ill-defined and unspecified causes of mortality” (ICD 10: R99) by the register, hospital records, laboratory and post-mortem results were accessed by the authors.

The “proportion of the data is made up of this classification” is already stated in the last sentence of the Results section:” Undefined causes of death were 22…”

3rd paragraph:Were these categories determined using a previously published methodology, or was it done in an adhoc way?
The categories were based on the main categories in the International Classification of Diseases (see earlier). See also reference Yu Y, Qin G, Cnattingius S, Gissler M, Olsen J, Zhao N, et al. (2016) Mortality in Children Aged 0-9 Years: A Nationwide Cohort Study from Three Nordic Countries. PLoS ONE 11(1): e0146669.

For further clarification, we added this reference to the paragraph.

5th paragraph: Since the entire study is about mortality rates, I would suggest explicitly writing how all rates were calculated (year, decade, group, sex, age), and which rates were used in what analysis.

We acknowledge this comment and have made changes to this paragraph accordingly. The paragraph is currently as follows:

Statistical analyses were done with R (ed 3.6.2) and R-studio (ed 1.2.5033).^9^ From the National Causes of Death register in Iceland and from population data from Statistics Iceland, death rate for each year, age groups and gender were calculated. This ratio was also calculated for the total mortality rate as well as for under five mortality rates. For analysing changes in death rates over the study period, Poisson regression (logarithmic transformed) was used with robust standard error. To calculate changes in death rates and gender ratios within the groups of causes of death as well as changes within age groups, a Chi-squared test was used comparing the first decade (1971-1980) to the last one (2009-2018). Statistical significance was set at p≤0·05 and a 95% confidence interval not crossing 1 for risk ratios.

Results:
1st paragraph: “Children aged 1-<5 years were 285 (14%, 58% boys), aged 5-<13 years old were 294 (15%, 63% boys) and 13-<18 years were 364 (18%, 72% boys) (Figure 1).” Is this referring to children who died?
Yes, the numbers in the results are referring to deceased children. We have added the word “Deceased” at the beginning of the sentence for clarification!

2nd paragraph: “Boys were 41% more likely to die during the whole period (CI: 0·51-0·67).” How was this calculation made?
This was obtained with the Poisson regression model. We have changed the sentence; it now reads as follows:

Boys were more likely to die during the whole period (RR: 1.45, CI: 1.33-1.59).

5th paragraph: This should be split into several paragraphs with different sections to guide the reader.
We have split this into several paragraphs (see also earlier comment).

Overall: I would suggest additional tables and/or figures to help cross walk the age and gender information. As it is, you cannot tell at what age boys and girls diverge in terms of suicide-based mortality. But this is important to a prevention measures.
We thank the reviewer for this suggestion. Accordingly, we have added another picture depicting this gender ratio changed figure 1 to show more age groups.

Figure 3: Gender ratios for all causes of childhood deaths and four different categories in Iceland during 1971-2018.

Figure 1: Why are there only three lines when 4 age classes are considered. Also, the age classes shown in the legend are the not the same as in the text.
We acknowledge this comment. Our objective with the figure was to emphasise the changes in <1 mortality rate. We have changed the figure to depict the four age groups as well as the changes in the total death rates.

Supplemental Table: This table appears to contain the same information as the Table 1 along with addition information. Why not just have 1 table?
The supplemental table is large. Our idea was to have Table I for the main readers and the supplemental table for those wanting more detailed information. We are willing to replace Table I with the supplemental table if the editors suggest that.

Discussion:

2nd paragraph: “The gender ratio revealed clearly a higher risk for boys than girls, most obvious in the oldest age group and more pronounced in earlier periods”. This seems contradictory.

This sentence has been changed:
The gender ratio clearly revealed a higher risk for boys than girls, most obvious in the oldest age group and more pronounced in the first decades of the study period.

4th paragraph: “In HIC, the number of prenatal births has increased”; do you mean premature births?
This has been changed to “premature”.

Overall: there are several assertions made without citations or data. For example “The reason for this gender difference may be due to behavioural differences as well as physiological, social and environmental factors. The decreasing gender ratio is primarily due to less fatal accidents among boys, probably through increased awareness and accident prevention.” While speculations about mechanisms of sex-based mortality differences or intervention success have to be made here, they should be made based on literature-based sources.

We thank the reviewer for this comment. The following references have been added to the manuscript:
For risk taking behaviour:

New reference:

Byrnes, James & Miller, David & Schafer, WD. (1999). Gender differences in risk taking: A meta-analysis. Psychol Bull. 125. 367-383. 10.1037//0033-2909.125.3.367.

Already in the reference list: Peden M, Oyegbite K, Ozanne-Smith J, et al. World report on child injury prevention. Geneva: World Health Organization; 2008.

For the discussion on malignancies: “As the incidence of malignancies in children has not decreased, this success must be attributed to improved cancer treatment as well as better supportive care, including infectious management.”

Already in the reference list:

Smith M.A., Altekruse S.F., Adamson P.C., Reaman G.H., Seibel N.L. Declining childhood and adolescent cancer mortality. Cancer. 2014. 120(16):2497-506.

Gatta G., Botta L., Rossi S., Aareleid T., Bielska-Lasota M., Clavel J., et al. Childhood cancer survival in Europe 1999–2007: results of EUROCARE-5—a population-based study. The Lancet Oncology. 2014. 15(1):35-47.

Reviewer #2: This is an important study on the changes in death rates in children in Iceland. My comments are as follows

We thank the reviewer for his encouraging review of the manuscript and constructive comments.

1. The manuscript is written in standard English but I do feel that some sentences are long or a little bit unclear and clarity could be improved throughout by a thorough edit.
We have made some adaptations to the text for clarification and an English speaking person has adjusted the language in several places.

2. Methods: Could 'cardio- vascular' just be 'cardiovascular'?
This has been changed throughout the manuscript

3. Methods: Could the authors provide a reference or further justification for why they grouped causes of death as they did?

The categories were based on the main categories in the International Classification of Disease. The smallest groups were put into other diseases. This has been clarifies slightly in the text

See also reference Yu Y, Qin G, Cnattingius S, Gissler M, Olsen J, Zhao N, et al. (2016) Mortality in Children Aged 0-9 Years: A Nationwide Cohort Study from Three Nordic Countries. PLoS ONE 11(1): e0146669.

For further clarification, we added this reference to the 3^rd^ paragraph of the methods section.

4. Results: Could the authors be consistent when they provide number and when they provide per cent? Sometimes they provide both and sometimes they just provide per cent. For example, in the first sentence, both numbers and percentages are provide, while in the second, only percentages are provided.
We acknowledge this comment and have adjusted the text to this suggestion throughout the manuscript.

5. Results: Paragraph 3. Sorry I may be misreading this, but I would have assumed the 95% CIs would be above 1 if boys were 41% more likely to die (1.59, 95% CI 1.51-1.67)? Could the authors put the RR with the CIs for clarity?

The text has been adapted according to this comment:

Boys were more likely to die during the whole period (RR: 1.45, CI: 1.33-1.59).

6. Results: I would prefer to see a p-value, rather than n.s. when the ratio is not significant.
This comment is absolutely correct. We have replaced n.s. with p=0.91

7. Results/Discussion: Why are avalanches not grouped with accidents?
We classified avaæamches separately as a natural disaster rather than accidents. We have adjusted the text according to this comment:

Results, final paragraph:

“…… and avalanches caused 16 deaths (11%), of those 12 occurred in the same year in two different avalanches.”

Discussion:

“In our study, sixteen children lost their lives in avalanches. These children were classified separately as natural disaster rather than accidents as they are different in nature and harder to prevent or predict.

8. Discussion: In the second paragraph, the authors say the gender ratio declined from 1.8 to 1.2. In the results, it says it declined from 1.6 to 1.1. Which is correct?

We acknowledge the reviewer for this comment and compliment him for the thorough reviewing. This was indeed a typing error on our behalf and has been corrected. The correct numbers are 1.6 to 1.2

9. Discussion: Second paragraph. The last sentence "This deserves special attention", leaves me asking 'why?' I would recommend the authors either delete or add more detail on why.
This paragraph has been rephrased and adjusted. It now reads as follows:

The gender ratio clearly revealed higher risk of death for boys than girls, most obvious in the adolescent age group and more pronounced in the first decades of the study period. These ratios have been described by others.^9,10^ This gender difference is less clear in under five mortality rates as has also been described by UNICEF in HIC.^1^ In our study, a decline in male/female ratio was seen from 1·6 to 1·2 and deserves special attention. The reason may be due to behavioural differences as well as physiological, social and environmental factors. This is reflected in the higher death rate for boys due to accidents. The decreasing gender ratio was in fact primarily driven by fewer fatal accidents among boys. These causes have been identified in several countries^8^. During the study period, several safety measures for accident prevention were implemented, especially regarding traffic safety. These safety measures as well as increased awareness are probably the main factors leading to the decreased childhood mortality due to accidents.

10. Discussion: Fourth paragraph. I got a little lost here. Weren't perinatal causes the most common cause of death? Could the authors clarify?
The paragraph currently reads as follows:

The highest risk of mortality was in the age group less than one year of age followed by the oldest age group, 13 to <18 years. This is comparable to other studies in HIC.^12,13^ The reason for the relative high death rate in the youngest group may be contributed to perinatal and neonatal events as well as congenital disease. In HIC, premature births have become more common, but the survival rate of premature babies has improved. This has also been found in Iceland^12^. Nevertheless, perinatal events may still cause some of these early deaths.^2,13,14^ The causes of death in the oldest age group are most often related to long term illnesses, accidents or suicide.

Reviewer #3: This manuscript presents interesting analyses of changes in death rates over time in Iceland. These analyses contain some valuable contributions, though I would suggest some changes to the analyses to make them more robust.
I also feel as though this manuscript could take the analyses further in the examples presented. Maybe this is just the epidemiologist in me, but, for instance, could the measles vaccination rates be included as a covariate in a model to determine if the rates are correlated with the declines in measles infections. For the decrease in accidents, maybe there were specific government campaigns to improving driving education or maybe just safer cars were released.
We thank the reviewer for this comment and interesting discussion. We do agree that epidemiological data in this context are interesting. However, with the small population of Iceland, the numbers are bound to be small. As an example, during the study period (started approximately ten years after the introduction of measles vaccine in Iceland), no child died of measles.

For the decrease in accidents, there were indeed several governmental actions as well as improved driving education and other societal and driving improvements. It is difficult to point out the most important factors. This is also the reason why multivariate analysis was not used. This study covers childhood death rates over a period of fifty years with a number of interventions and societal changes occurring in the period (new treatment options, laws and regulations for traffic safety, improved diagnostics for various diseases etc.). In addition, these factors weigh differently on the various causes of death and changes with time. However, the discussion has been adapted for further clarification of the factors mentioned.

1. For the Poisson distribution, the mean and variance are the same parameter, i.e., the mean equals the variance. That can be a very poor assumption in regression models and I would strongly urge you to try a quasipossion or negative binomial model. Those distributions decouple the mean and variance and allow for the variance to be estimated independent of the mean.

2. My understanding is that you've modeled the rates. I would suggest trying to model the death counts instead of the rates with the population as an offset. This is a more natural way to model this, and you can obtain the death rates from the model and convert them to deaths per 1000.

3. I also wasn't sure why a Chi-squared test was used especially after you've been working with a model that is testing trends over time. You could utilize the model to estimate the changes from the start of the interval to the end, probably with contrast statements.

4. In the results, I would be a little more explicit about some of the sample sizes presented. The sentence about deaths from accidents is great, but it's hard to tell what the time frame is for the other diseases.

We do acknowledge this interesting discussion and suggestions regarding the statistical methods. This has again been discussed in the study group as well as with our statistical consultant from the University of Iceland. We do agree with the reviewer that different statistical methods may have been applied. However, this also has some limitations. As mentioned before, the Icelandic population is small and the groups, especially the subgroups contain low numbers of individuals. We felt that the subgroups were too small to be able to use the Poisson regression to estimate the change correctly and the goodness of fit test came out invalid. Using ordinal logistic regression gave similar results.

Our first reviewer found “the statistical methods chosen to be reasonable”. Nevertheless, some minor changes have been made to the material and methods section and the Results.

We certainly hope that the reviewer finds this sufficient!
