# Supplementary material for: Decreasing death rates and causes of death in Icelandic children—A longitudinal analysis
Source: PLoS One. 2021 Sep 30;16(9):e0257536. doi: 10.1371/journal.pone.0257536 (PMC8483359; doi:10.1371/journal.pone.0257536)
Supplement: Attachment — Submitted filename: Decreasing death rates in children - Resp to reviewers II.docx [file pone.0257536.s003.docx]

PONE-D-20-37583R1
Decreasing death rates and causes of death in Icelandic children - A longitudinal analysis
PLOS ONE

Dear Dr. Haraldsson,

Thank you for submitting your manuscript to PLOS ONE. After careful consideration, we feel that it has merit but does not fully meet PLOS ONE’s publication criteria as it currently stands. Therefore, we invite you to submit a revised version of the manuscript that addresses the points raised during the review process.


Specifically ,please address all comments made by reviewer #1

Please submit your revised manuscript by Aug 07 2021 11:59PM. If you will need more time than this to complete your revisions, please reply to this message or contact the journal office at [plosone@plos.org](mailto:plosone@plos.org). When you're ready to submit your revision, log on to <https://www.editorialmanager.com/pone/> and select the 'Submissions Needing Revision' folder to locate your manuscript file.

Please include the following items when submitting your revised manuscript:

- A rebuttal letter that responds to each point raised by the academic editor and reviewer(s). You should upload this letter as a separate file labeled 'Response to Reviewers'.
- A marked-up copy of your manuscript that highlights changes made to the original version. You should upload this as a separate file labeled 'Revised Manuscript with Track Changes'.
- An unmarked version of your revised paper without tracked changes. You should upload this as a separate file labeled 'Manuscript'.

If you would like to make changes to your financial disclosure, please include your updated statement in your cover letter. Guidelines for resubmitting your figure files are available below the reviewer comments at the end of this letter.

If applicable, we recommend that you deposit your laboratory protocols in protocols.io to enhance the reproducibility of your results. Protocols.io assigns your protocol its own identifier (DOI) so that it can be cited independently in the future. For instructions see: <http://journals.plos.org/plosone/s/submission-guidelines#loc-laboratory-protocols>. Additionally, PLOS ONE offers an option for publishing peer-reviewed Lab Protocol articles, which describe protocols hosted on protocols.io. Read more information on sharing protocols at <https://plos.org/protocols?utm_medium=editorial-email&utm_source=authorletters&utm_campaign=protocols>.

We look forward to receiving your revised manuscript.

Kind regards,

Amir Radfar, MD,MPH,MSc,DHSc
Academic Editor
PLOS ONE

[Note: HTML markup is below. Please do not edit.]

Reviewers' comments:

Reviewer's Responses to Questions

**Comments to the Author**

1. If the authors have adequately addressed your comments raised in a previous round of review and you feel that this manuscript is now acceptable for publication, you may indicate that here to bypass the “Comments to the Author” section, enter your conflict of interest statement in the “Confidential to Editor” section, and submit your "Accept" recommendation.

Reviewer #1: (No Response)

Reviewer #3: All comments have been addressed

2. Is the manuscript technically sound, and do the data support the conclusions?

The manuscript must describe a technically sound piece of scientific research with data that supports the conclusions. Experiments must have been conducted rigorously, with appropriate controls, replication, and sample sizes. The conclusions must be drawn appropriately based on the data presented.

Reviewer #1: Yes

Reviewer #3: (No Response)

3. Has the statistical analysis been performed appropriately and rigorously?

Reviewer #1: I Don't Know

Reviewer #3: (No Response)

4. Have the authors made all data underlying the findings in their manuscript fully available?

The [PLOS Data policy](http://www.plosone.org/static/policies.action#sharing) requires authors to make all data underlying the findings described in their manuscript fully available without restriction, with rare exception (please refer to the Data Availability Statement in the manuscript PDF file). The data should be provided as part of the manuscript or its supporting information, or deposited to a public repository. For example, in addition to summary statistics, the data points behind means, medians and variance measures should be available. If there are restrictions on publicly sharing data—e.g. participant privacy or use of data from a third party—those must be specified.

Reviewer #1: Yes

Reviewer #3: (No Response)

5. Is the manuscript presented in an intelligible fashion and written in standard English?

PLOS ONE does not copyedit accepted manuscripts, so the language in submitted articles must be clear, correct, and unambiguous. Any typographical or grammatical errors should be corrected at revision, so please note any specific errors here.

Reviewer #1: Yes

Reviewer #3: (No Response)

6. Review Comments to the Author

Please use the space provided to explain your answers to the questions above. You may also include additional comments for the author, including concerns about dual publication, research ethics, or publication ethics. (Please upload your review as an attachment if it exceeds 20,000 characters)

Reviewer #1: The authors have reasonably responded to most of my comments and have significantly improved their manuscript. However, there are still some import issues that need to be addressed before I can recommend this manuscript for publication.

We like to thank the reviewer for thorough reviewing of the manuscript and constructive and adequate comments.

INTRODUCTION
The end of the second paragraph and the last paragraphs are a bit confusing. The author’s state that the main hypothesis that childhood death rates are decreasing due to fewer fatal infections. But then the author’s say that the underlying hypothesis is that death rates are declining. I think that what the authors are aiming to say is that the data show that death rates have fallen over the previous 5 decades and it is a hypothesis is that these declines are primarily driven by decreases in fatal infections, childhood accidents, and neonatal mortality. If this is correct, this should be clarified.

We acknowledge this comment and have adapted the manuscript accordingly. To the second paragraph we have added the sentence This data show that death rates have fallen in Iceland over the last decades and the last century.

We have changed the last paragraph according to the reviewer’s suggestion. It currently reads as follows:

The aim of the current study was to describe changes in childhood death rates in Iceland and evaluate causes of death over almost five decades. The underlying hypothesis was that the declining death rates are primarily driven by decreases in fatal infections, childhood accidents, and neonatal mortality, especially in the under-five age group. From the results we attempt to determine specific risk factors and identify potential interventions to further prevent childhood deaths.

METHODS
In the statistical analysis section, the authors say they have stated how rates were calculated, but they only say in the methods that “death rate for each year, age groups and gender were calculated”. In the results, it can be seen that rates are listed per 1000 children. However, providing a simple equation for rate calculation, and/or stating that they are rates of death per thousand children in the methods section would be helpful to clear up ambiguity.

We thank the reviewer for pointing this out to us and have added the following to the methods section. We have moved the sentence on the number of living children from the second paragraph to this one and added information to the text. The paragraph reads as follows:

In the current study, causes of death in children (from birth until their 18th birthday) were collected from The National Causes of Death register in Iceland. Death rates were calculated and classified according to year of death, age-groups and gender. Death rates were calculated and given as rates of death per thousand children. Information on number of living children each year and stratified to age and gender was obtained from Statistics Iceland (statice.is).

RESULTS
There is still uncertainty about the Poisson regression model that was applied to the data. Was a separate regression model fit for each group, or was a single model used with age and gender groups as factors? Which factors were significant and which factors weren’t? If multiple models were applied, was there a correction for multiple p-values. These issues could be addressed by simply reporting the terms of the model used in the text. In addition, PLoSOne guidelines for regression analyses suggest that they should be included, at least as part of the supplementary data:
Regression analyses. Include the full results of any regression analysis performed as a supplementary file. Include all estimated regression coefficients, their standard error, p-values, and confidence intervals, as well as the measures of goodness of fit.
On the same topic, providing the details of all the Chi-square analyses performed would also be helpful to fully appreciate the scope of the analysis that the authors conducted.

We thank the reviewer for his detailed and constructive comments on the statistical presentation.

There were indeed two regression models used, one for all children 0-18 years old and another one for children <5 years of age. The regression model for children of all ages was done with an interaction variable between genders. This variable was insignificant in the model for children under the age of 5 and was not included (the html files will indeed be uploaded). ANOVA was done to compare the results with and without that variable. A html file with the chi square analyses as will be uploaded as suggested.

We have made some adaptations to the test to clarify this. However, to make the manuscript not to complicated to read, we have kept these changes to the minimum. The following has been added to the manuscript

Two regression models were applied, one for all children 0-18 years old and another one for children under 5 years of age. The regression model for children of all ages was done with an interaction variable between genders. ANOVA was done to compare the results with and without that variable.

In addition, html files for two Poisson Regressions as well as a file with the underlying data and file with the ChiSquare analysis will be uploaded.

DISCUSSION
The statement that “the decrease was continuous over the period” does not appear to be true. There is clear variation from year to year with increases in some groups occurring several years in a row (e.g. Figure 1, group 13-18, 1982-1987). However, the trend appears to be consistently negative over the period, which is what the Poisson regression (I think?) showed.

This has been corrected and the words “the decrease was continuous over the period” have been replaced with “the trend appears to be consistently negative over the period”

Did the authors quantify that “The decreasing gender ratio was in fact primarily driven by fewer fatal accidents among boys”? While it is mentioned in the results that there was a dramatic decrease in the boys:girls death ratio of accidents, and figure 3 is provided to show this, I don’t see any sort of quantification of the contribution of accidents, relative to other causes of death, to differences in boy:girl death ratios between the two decades of comparison.

We thank the reviewer for this comment.

In the result section we already mention the change in death rate per 1000 children due to accidents over the study period (The gender ratio in accidents (boys:girls) was 3.42 (p<0.0001) in the first decade but 0.96 (p=0.91) in the last decade). Part of this data can be seen in the table. However, in order not to make the data to complicated, we did not compare the ten groups against each other. Nevertheless, these number for all groups can be seen in the table.

We rephrased the sentence, it now reads as follows:

The changes in gender ratios were clearly reflected in the decreasing death ratio caused by accidents among boys.

In addition, figure 3 is at a very low resolution, making the y-axis largely unreadable. Although I can see that accidents are the only cause of death for which male and female death rates (maybe counts?) do not obviously overlap, the y-axes are clearly on different scales. A quantification here would make the author’s argument on this point stronger.

We thank the author for pointing this out and apologize for the quality of the figure. This has been corrected.

In the sentence “The reason for the relative high death rate in the youngest group may be contributed to perinatal and neonatal events as well as congenital disease”, I think the authors might want to use the word “attributed” instead of “contributed”.

Thank you for this remark, the wording has been corrected.

For the statement “Traffic accidents were the most common cause of accidents and accounted for more than half of the fatal accidents. In the younger age groups, most victims were pedestrians, but older children were more often car passengers”, I don’t see where this is shown, as the supplementary table is not stratified by age. Since there are no limits on the size of supplementary files, it seems reasonable to include this information if it is reported in the text. Also, is the implication here that older boys would have higher traffic mortality as the drivers of cars than girls? If so, that should be stated.

We thank the reviewer for this comment (and compliment him/her for the observation). The data for this is available. However, in order not to overload the manuscript with data and tables, we decided to leave out the sentence “In the younger age groups, most victims were pedestrians, but older children were more often car passengers”

In the sentence “The available data may not always be sufficient to distinguish between accidents e.g. by drug overdose and suicides”, I believe that the authors need to add a parentheses: “…accidents (e.g., by drug overdose) and suicides”.

Parenthesis have been applied

Reviewer #3: (No Response)

7. PLOS authors have the option to publish the peer review history of their article ([what does this mean?](https://journals.plos.org/plosone/s/editorial-and-peer-review-process#loc-peer-review-history)). If published, this will include your full peer review and any attached files.


If you choose “no”, your identity will remain anonymous but your review may still be made public.


**Do you want your identity to be public for this peer review?** For information about this choice, including consent withdrawal, please see our [Privacy Policy](https://www.plos.org/privacy-policy).

Reviewer #1: No

Reviewer #3: No

[NOTE: If reviewer comments were submitted as an attachment file, they will be attached to this email and accessible via the submission site. Please log into your account, locate the manuscript record, and check for the action link "View Attachments". If this link does not appear, there are no attachment files.]

While revising your submission, please upload your figure files to the Preflight Analysis and Conversion Engine (PACE) digital diagnostic tool, <https://pacev2.apexcovantage.com/>. PACE helps ensure that figures meet PLOS requirements. To use PACE, you must first register as a user. Registration is free. Then, login and navigate to the UPLOAD tab, where you will find detailed instructions on how to use the tool. If you encounter any issues or have any questions when using PACE, please email PLOS at [figures@plos.org](mailto:figures@plos.org). Please note that Supporting Information files do not need this step.
